# Supplementary material for: Cryo-electron microscopy reveals two distinct type IV pili assembled by the same bacterium
Source: Nat Commun. 2020 May 6;11:2231. doi: 10.1038/s41467-020-15650-w (PMC7203116; doi:10.1038/s41467-020-15650-w)
Supplement: Supplementary file 1 — Supplementary Information [file 41467_2020_15650_MOESM1_ESM.pdf]

Supplementary Information

Cryo-electron microscopy reveals two distinct type IV pili assembled by the same bacterium

Neuhaus *et al*.

Supplementary Figures

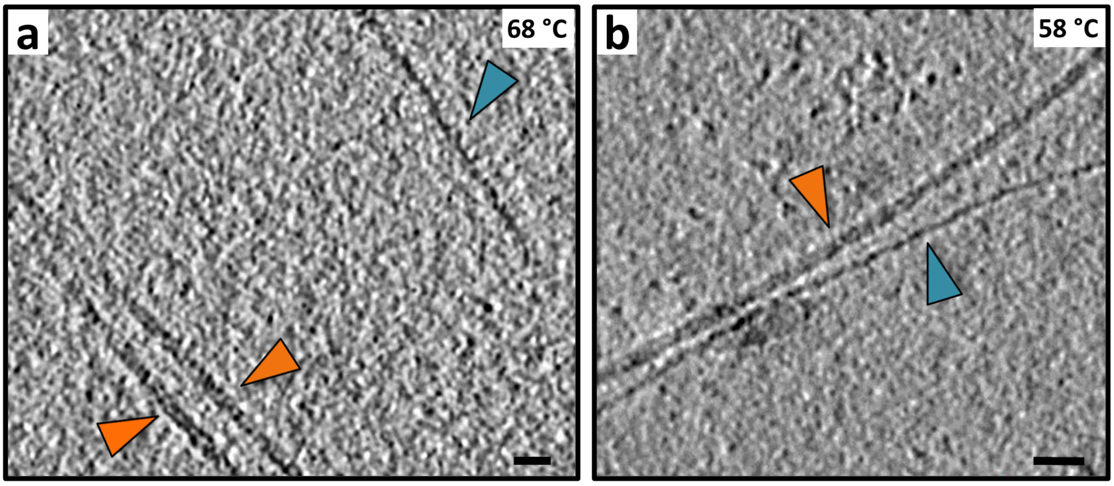

Supplementary Figure 1: Purified pilus preparations contain both wide and narrow forms

a, b, Tomographic slices through isolated pili from *T. thermophilus* grown at 68 °C (**a**) or 58 °C (**b**) show both wide (orange arrowheads) and narrow (teal arrowhead) pili. Scale bars, 20 nm.

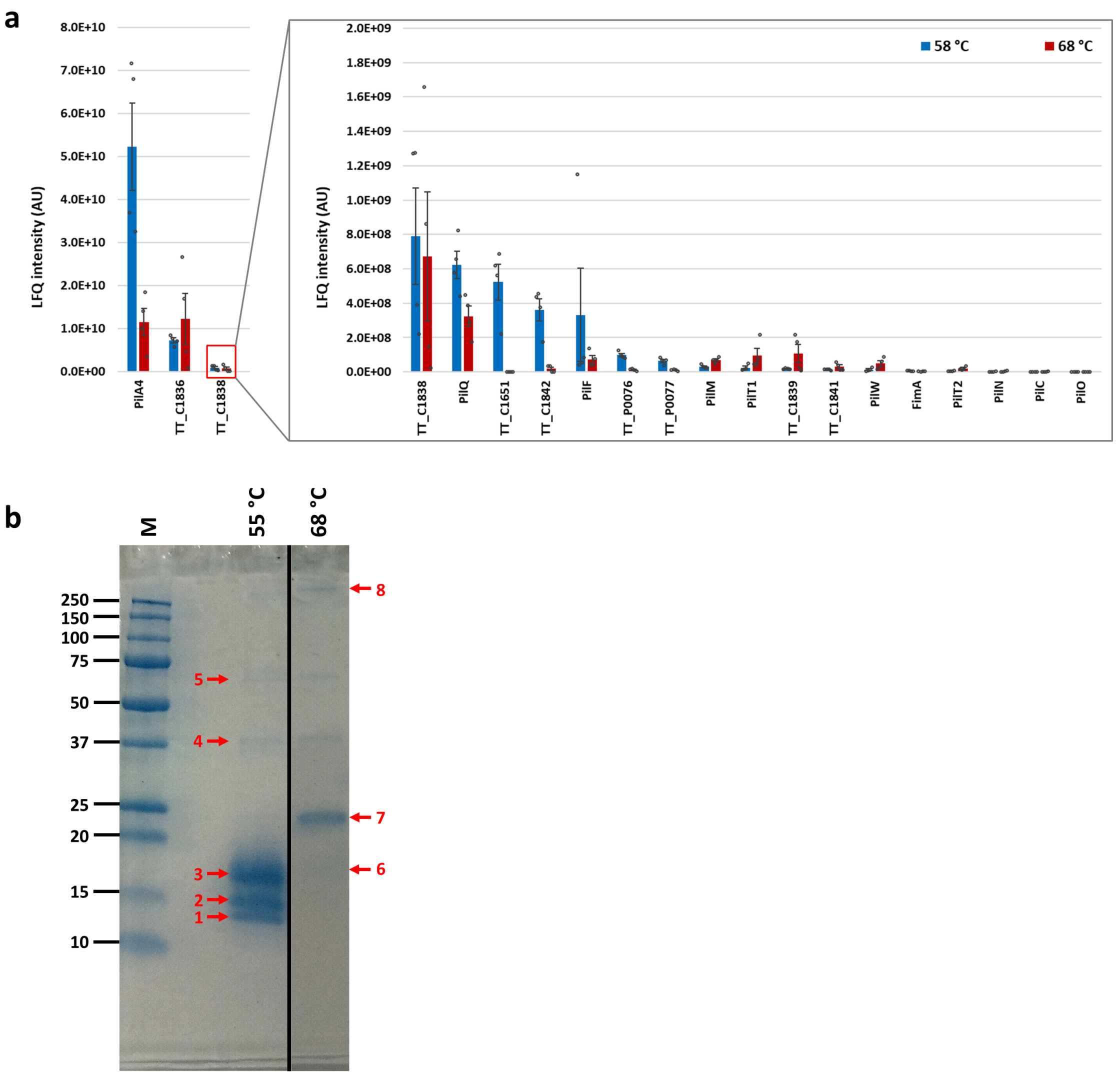

Supplementary Figure 2: Mass spectrometry of isolated pili

a, LFQ intensity analysis showing the relative abundance of different pilin-like and pilus associated proteins at 58 °C and 68 °C. Data are presented as mean values +/- SEM. Differential abundance of proteins (detected in at least 3 of 4 replicates in each condition) was analysed using a two-sided t-test with an FDR of 0.01 and s0 = 0.05.

b, Gel-based MS of isolated pili. Pili isolated from cells grown at 55 °C or 68 °C were purified and separated by SDS-PAGE. The numbered bands were analysed by MS and the most abundant proteins in each band are listed in Supplementary Table 1.

Source data are shown in Supplementary Figure 10 (panel b) and in a Source Data file (both panels).

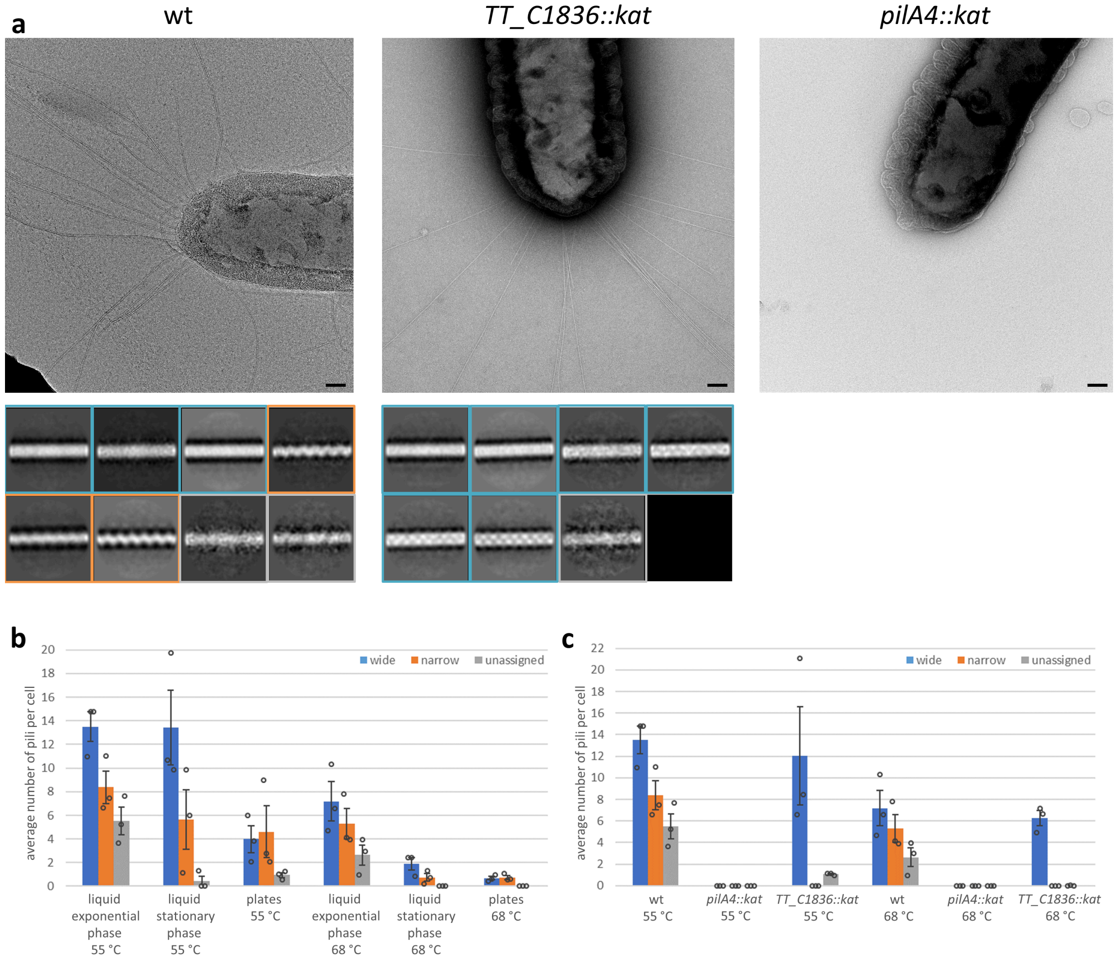

Supplementary Figure 3: Analysis of the abundance of wide and narrow pili

a, Top panels, representative electron micrographs of negatively stained *T. thermophilus* cells show long flexible filaments for wild-type and *TT_C1836::kat* cells. Scale bars, 100 nm. Bottom panels, examples of 2D class averages of pili from electron micrographs of whole cells. Class averages were assigned to wide pili (teal boxes) or narrow pili (orange boxes). Some filaments could not be assigned to classes reliably (grey boxes). The number of particles in each class was used for further evaluation in **b** and **c**. Box size, 35 nm.

b, Number and type of pilus assembled for wild-type cells under different growth conditions. Data are presented as mean values +/- SEM.

c, Number and type of pilus analysed for mutants grown to exponential phase. Data are presented as mean values +/- SEM.

Source data for panels b-c are provided in a Source Data file.

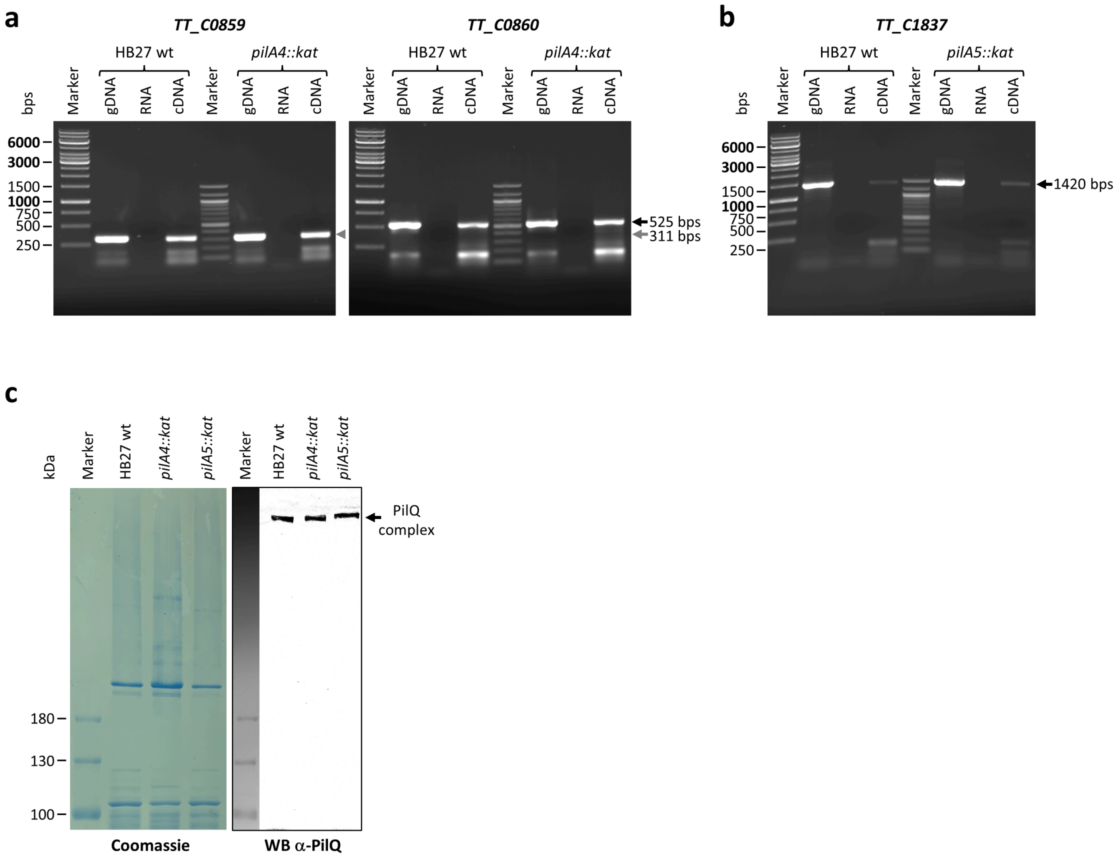

Supplementary Figure 4: There are no polar effects of pilin deletions on nearby genes and PilQ complex formation

a, b, Transcript analyses of genes located downstream of *pilA4* and *pilA5* in wild-type cells and in *pilA4::kat* (**a**) or *pilA5::kat* (**b**) mutants. For *pilA4::kat,* primers for *TT_C0859* and *TT_C0860* were used; for *pilA5::kat*, primers binding *TT_C1837* were used. For the positive control, amplification was performed on 500 ng of genomic DNA (gDNA), and as a negative control, 350 ng DNase treated RNA was used. Amplified PCR products corresponding to the expected DNA fragments are marked with arrows.

c, SDS-PAGE gel showing the detection of PilQ complexes in total membranes of wild-type cells, *pilA4::kat* and *pilA5::kat* mutants. The PilQ complex was detected by Western blot analysis (right) using a polyclonal PilQ antibody. SDS-PAGE and Western blot were derived from the same sample.

Source data are shown in Supplementary Figure 10 and provided in a Source Data file.

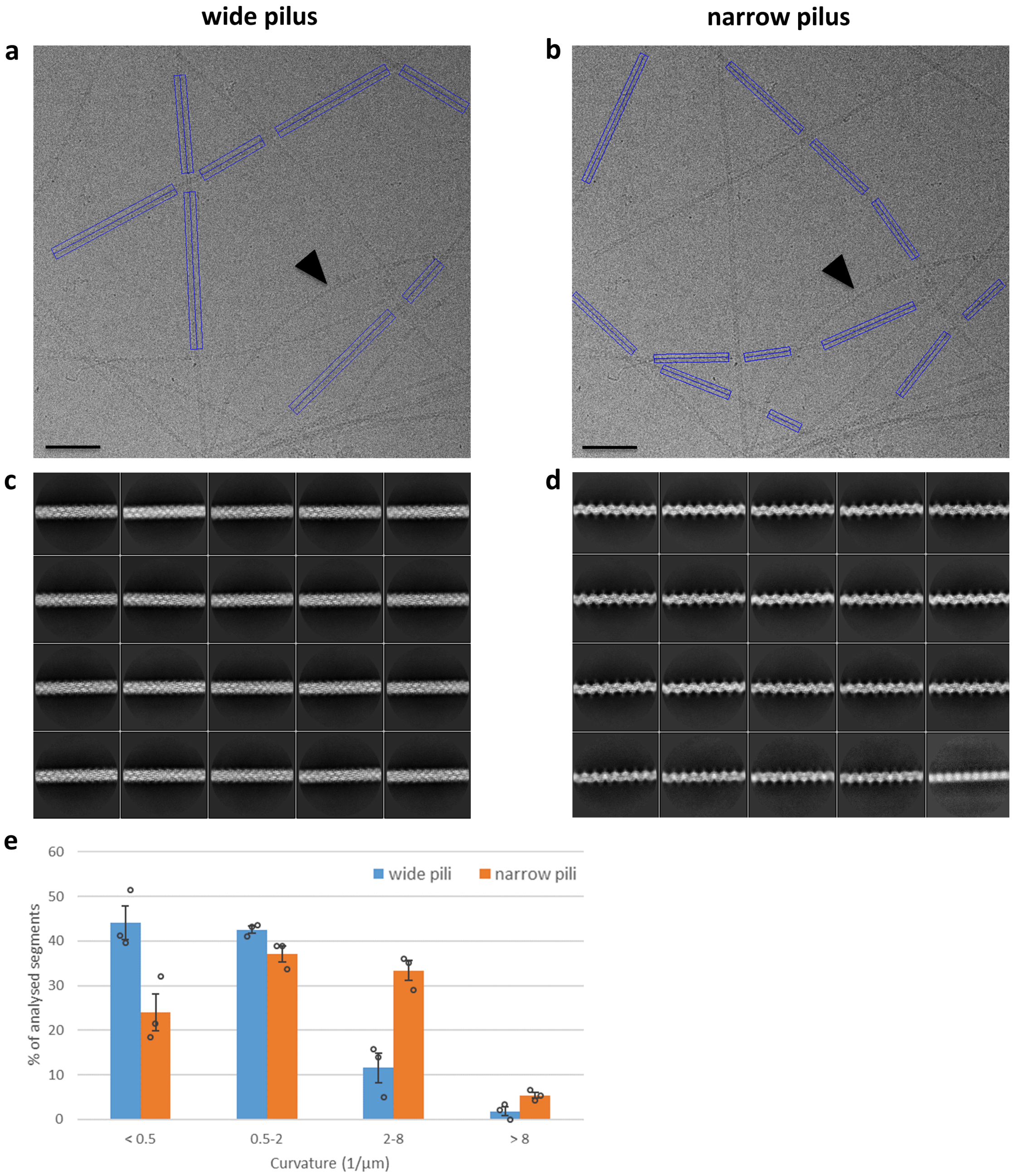

Supplementary Figure 5: Raw CryoEM data and 2D classes

a, b, CryoEM micrograph with selected wide (**a**) and narrow (**b**) pili. The arrowhead indicates a narrow pilus with a high degree of curvature that was often observed for this type of filament. Scale bars, 50 nm.

c, d, 2D classes of wide (**c**) and narrow (**d**) pili. Box size, 40 nm.

e, Bar chart showing the curvature distribution of wide and narrow pili obtained from cryoEM micrographs. Data are presented as mean values +/- SEM.

Source data for panel e are provided in a Source Data file.

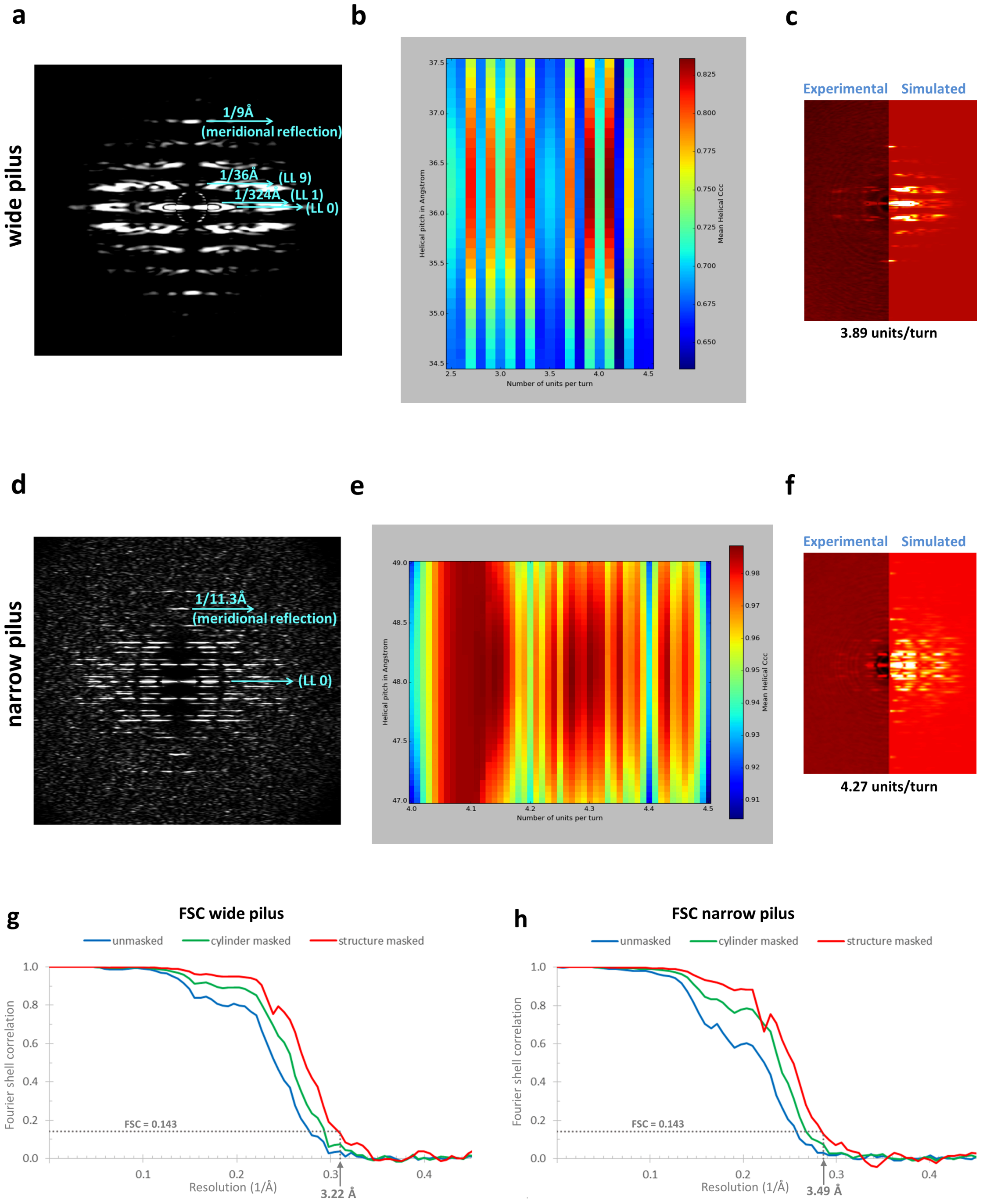

Supplementary Figure 6: Helical symmetry determination using SPRING

a, d, B-factor enhanced power spectra from 2D classes of wide **(a)** and narrow **(d)** pili. The meridional reflections and selected layer lines are labelled. LL, layer line.

b, e, SEGCLASSRECONSTRUCT (SPRING) results for wide (**b**) and narrow (**e**) pili. For wide pili a high correlation was observed for 4.1 and 3.89 subunits per turn and a helical pitch of ~36.3 Å (**b**). For narrow pili a high correlation was observed for 4.11, 4.14, 4.27 or 4.30 subunits per turn and a helical pitch of ~48.1 Å (**e**).

c, f, Agreement between layer line positions in experimental and simulated Fourier transforms for wide (**c**) and narrow (**f**) pili.

g, h, FSC curves for wide (**g**) and narrow (**h**) pili. The calculated final maps were determined at 3.22 Å resolution from 65,656 segments (196,968 asymmetric units) for wide filaments (**g**), and 3.49 Å from 51,301 segments (153,903 asymmetric units) for narrow filaments (**h**) using Fourier shell correlation (structure-masked, 0.143 cut-off). Blue curve, unmasked FSC; green curve, cylinder-masked FSC; red curve, structure-masked FSC.

Source data for panels g-h are provided in a Source Data file.

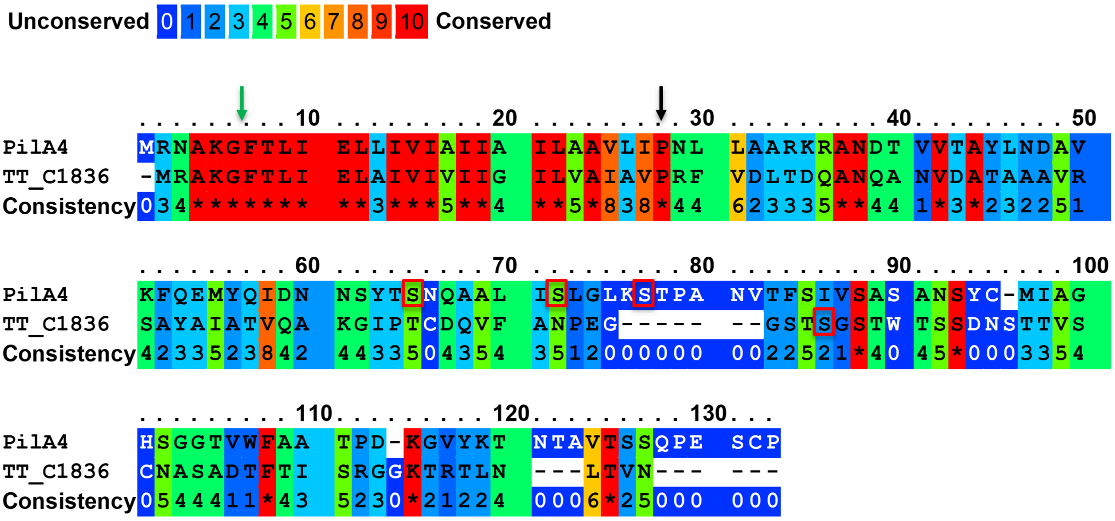

Supplementary Figure 7: Sequence alignment between PilA4 and TT_C1836

The cleavage site of prepilin peptidase (green arrow), the conserved Pro22 (black arrow) and glycosylation sites (red boxes) are shown.

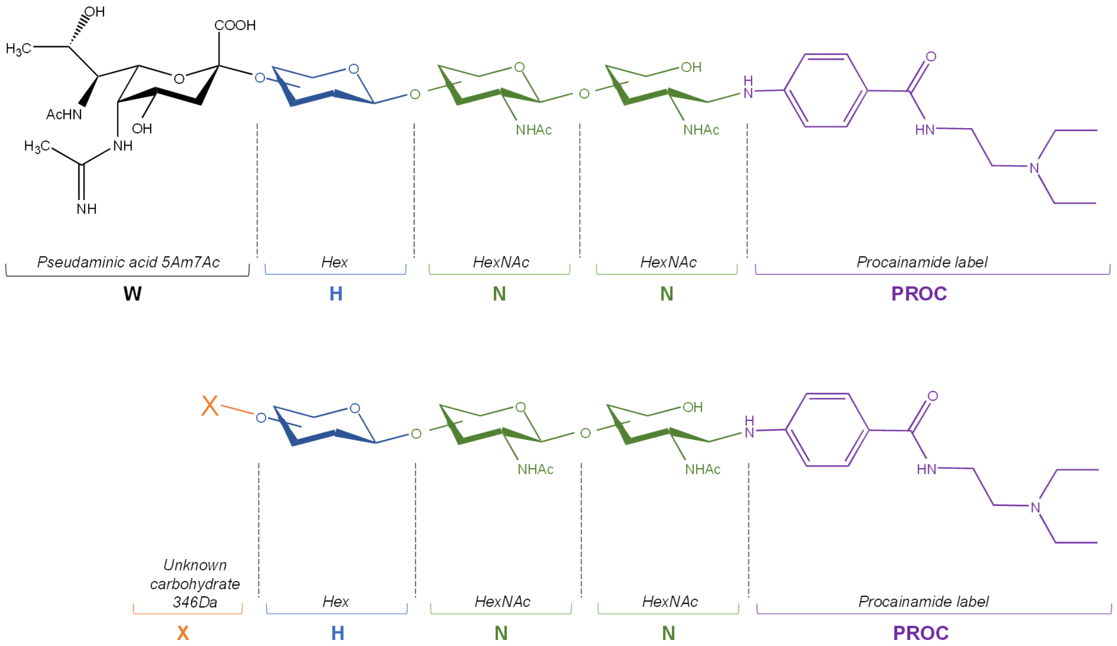

Supplementary Figure 8: Structures of the most abundant glycans

Structures of the two most abundant glycans W-H-N-N-PROC and X-H-N-N-PROC as identified by MS and MS/MS fragmentation. The procainamide label is attached to the reducing end of the glycan. H, Hex, hexose; N, HexNAc, N-acetylhexosamine; W, Pseudaminic acid derivative 5Am7Ac (7-Acetamido-5-acetimidoyl-3,5,7,9-tetradeoxy-L-glycero-L-manno-nonulosonic acid); X, unknown and previously unreported monosaccharides with a molecular mass of 346 Da; PROC, procainamide.

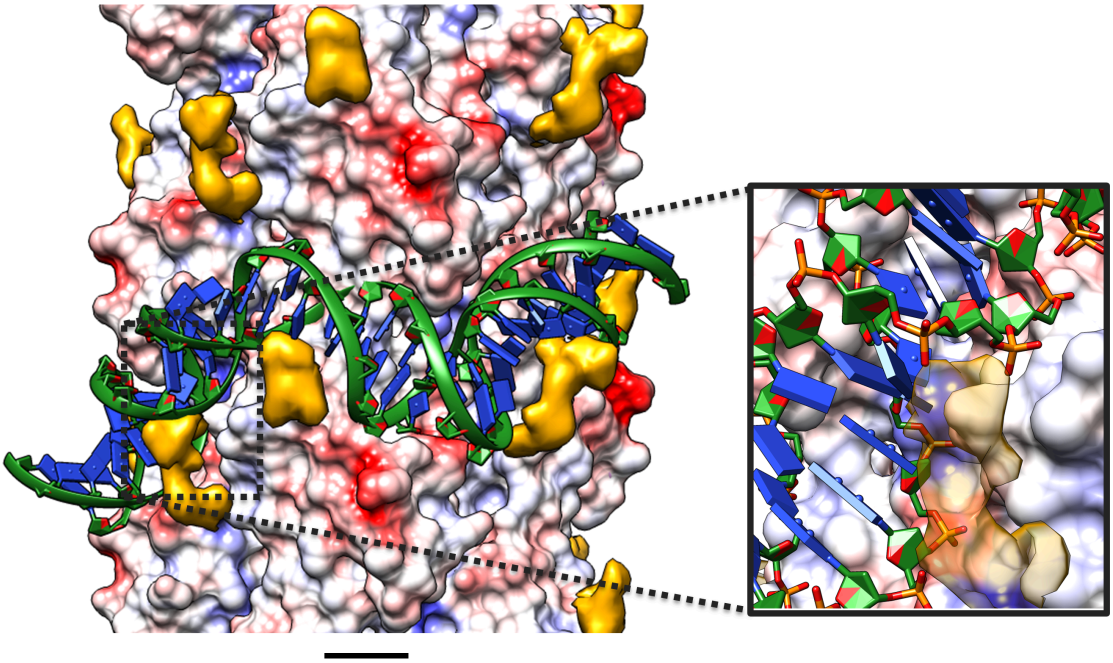

Supplementary Figure 9: Model of DNA bound to wide pili

A double stranded DNA molecule (green) is modelled around a wide pilus shown in surface charge representation (negative charges, red; positive charges, blue). The DNA backbone fits neatly into the positively charged groove of the PilA4 filament (inset). Post-translational modifications are shown in yellow (transparent yellow in inset). Scale bar, 10 Å.

Supplementary Figure 10: Uncropped original data images

Figure 5c

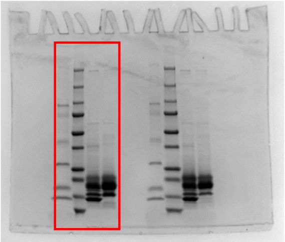

Coomassie
(coloured using GIMP)

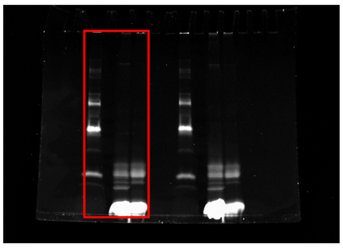

Glycoprotein stain

Figure 5d

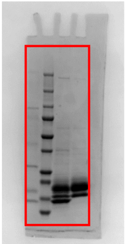

Coomassie
(coloured using GIMP)

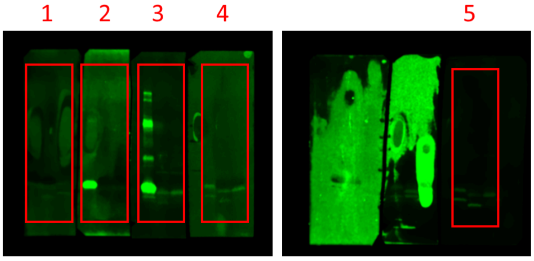

Lectin analysis 1
1, UEA1 (mirrored); 2, WGA; 3, ConA; 4, SBA; 5, DBA

Figure 6a

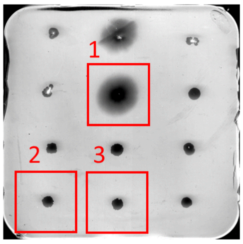

Twitching plate 68 °C
1, wt; 2, *pilA4::kat*;
3, *pilA5::kat*

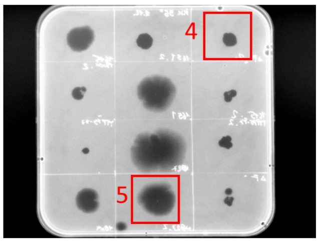

Twitching plate 55 °C
4, *pilA4::kat*; 5, wt

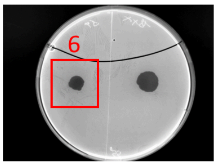

Twitching plate 55 °C
6, *pilA5::kat*

Supplementary Figure 2b

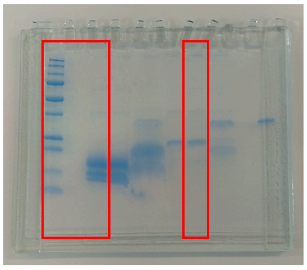

Coomassie

Supplementary Figure 4a

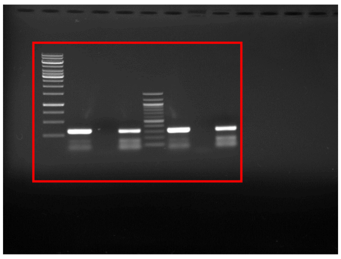

TT_C0859

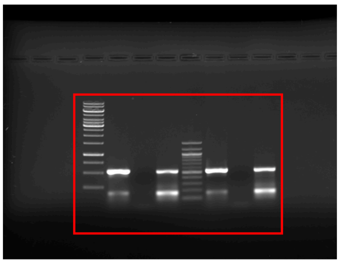

TT_C0860

Supplementary Figure 4b

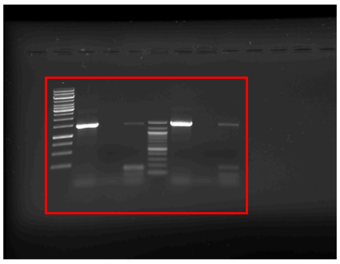

TT_C1837

Supplementary Figure 4c

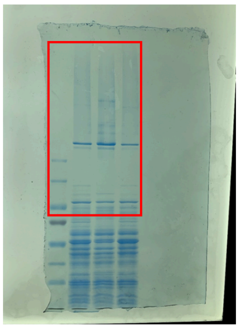

Coomassie

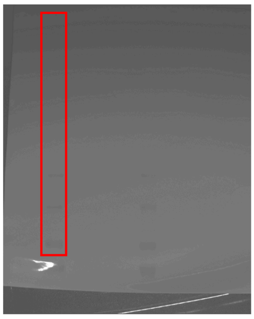

Western blot marker

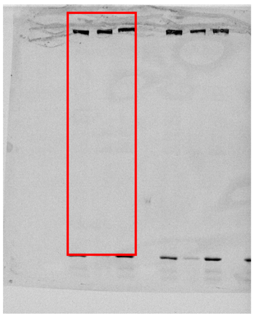

Western blot antibody

Band		Accession	Description	Score	Coverage	# Peptides	# PSMs	Area	# AAs	MW [kDa]
**Band 1**	1	Q72GL2	TT_C1836	291.44	27.59	3	63	6.194E7	116	12.0
	2	Q72JC0	PilA4	243.36	73.28	6	63	1.483E9	131	13.9
	3	Q72KA8	TT_C0539	71.52	67.15	9	21	6.243E7	137	14.6
**Band 2**	1	Q72JC0	PilA4	430.74	73.28	6	121	1.075E10	131	13.9
	2	Q72GL2	TT_C1836	282.84	27.59	3	70	3.161E9	116	12.0
	3	Q72GK9	TT_C1839	98.70	75.33	10	27	8.063E7	150	16.6
**Band 3**	1	Q72JC0	PilA4	378.49	73.28	7	103	1.773E10	131	13.9
	2	Q72GL2	TT_C1836	48.31	33.62	4	13	5.566E8	116	12.0
	3	Q72JD8	TT_C0834	35.21	30.36	6	9	3.844E7	224	23.7
**Band 4**	1	Q72J15	TT_C0966	371.61	83.72	29	105	5.358E8	387	41.6
	2	Q72K21	TT_C0597	170.71	65.90	25	56	1.475E8	390	42.5
	3	Q72LP7	TT_C0011	113.46	53.00	17	32	3.841E7	417	45.3
**Band 5**	1	Q72I61	TT_C1271	296.92	48.55	41	112	4.161E8	622	70.2
	2	Q746H7	TT_P0078	256.88	46.60	29	76	6.041E7	633	69.1
	3	Q72L70	TT_C0193	128.04	45.01	32	44	2.737E6	822	90.0
**Band 6**	1	Q72JC0	PilA4	156.59	25.95	4	44	4.258E8	131	13.9
	2	Q72JD8	TT_C0834	45.19	34.38	6	11	3.350E7	224	23.7
	3	Q72KR8	yceI	35.58	51.69	9	16	1.288E7	178	19.8
**Band 7**	1	Q72JD8	TT_C0834	499.51	47.32	10	128	9.016E9	224	23.7
	2	Q72JG5	TT_C0807	90.69	59.84	13	26	2.759E7	254	28.4
	3	Q72KM3	TT_C0384	50.47	55.80	11	18	5.221E7	224	24.2
**Band 8**	1	Q72HF5	TT_C1532	441.67	55.49	42	131	3.943E8	948	100.6
	2	Q72J15	TT_C0966	114.66	45.74	16	33	6.250E6	387	41.6
	3	Q72HL5	TT_C1472	101.66	35.75	12	30	5.943E7	372	40.0

Supplementary Table 1: Proteomics of isolated pili samples (SDS-PAGE)

Results of gel-based MS (shown in Supplementary Figure 2b). For each band the proteins with the highest scores are listed. PilA4 and TT_C1836 are highlighted in green and blue, respectively.

	#1 PilA4 (EMD-10647) (PDB 6XXD)	#2 PilA5 (TT_C1836) (EMD-10648) (PDB 6XXE)
**Data collection and processing**		
Magnification	130 kx	Same dataset as #1
Voltage (kV)	300	Same dataset as #1
Electron exposure (e–/Å^2)	47.56	Same dataset as #1
Defocus range (µm)	1.5-4	Same dataset as #1
Pixel size (Å)	1.048	Same dataset as #1
Symmetry imposed	helical	helical
Initial particle images (no.)	83,198 (3 asym. su/particle)	77,985 (3 asym. su/particle)
Final particle images (no.)	65,656 (3 asym. su/particle)	51,301 (3 asym. su/particle)
Map resolution (Å)	3.22	3.49
FSC threshold	0.143	0.143
**Refinement**		
Initial model used (PDB code)	*de novo*	*de novo*
Model resolution (Å)	3.3	3.6
FSC threshold	0.5	0.5
Map sharpening *B* factor (Å^2)	-60	-60
Model composition	16 identical subunits	16 identical subunits
Non-hydrogen atoms	14,896	12,816
Protein residues	2,000	1,776
Ligands	0	0
B factors (Å^2)		
Protein	38.85 (chain A only)	52.27 (chain A only)
Ligand		
R.m.s. deviations		
Bond lengths (Å)	0.006	0.006
Bond angles (°)	0.882	1.014
Validation		
MolProbity score	1.41 (all chains) 0.89 (chain A only)	2.18 (all chains) 1.80 (chain A only)
Clashscore	2.40 (all chains) 0.0 (chain A only)	6.11 (all chains) 1.87 (chain A only)
Poor rotamers (%)	0	2.3
Ramachandran plot		
Favored (%)	94.3	89.0
Allowed (%)	5.7	11.0
Disallowed (%)	0	0

Supplementary Table 2: Cryo-EM data collection, refinement and validation statistics

Both final models contain 16 identical subunits by imposing the respective helical symmetry to the central subunit (chain A). Therefore some values are only available for chain A.

PilA4

distance between Cα and N-terminus Cα (Angstrom):

amino acid

		E5	D33	D42	E48	D53	D106	E122	C-terminus
	+2	>20	>20	>20	>20	>20	>20	>20	>20
	+1	13.0	>20	>20	>20	>20	>20	>20	>20
	0	6.7	>20	>20	>20	>20	>20	>20	>20
	-1	9.9	>20	>20	>20	>20	>20	>20	>20
	-2	16.1	>20	>20	>20	>20	>20	>20	>20
subunit	-3	>20	>20	>20	>20	>20	>20	>20	>20
	-4	>20	>20	>20	>20	>20	>20	>20	>20
	-5	>20	15.7	18.2	>20	>20	>20	>20	>20
	-6	>20	14.1	11.4	18.5	>20	19.9	>20	>20
	-7	>20	>20	15.6	16.7	>20	17.5	>20	>20
	-8	>20	>20	>20	17.7	>20	>20	>20	>20
	-9	>20	>20	>20	>20	19.1	>20	>20	>20
	-10	>20	>20	>20	>20	>20	>20	>20	>20

distance between specified atoms (Angstrom):

Phe1@N (Subunit 0) - Glu5@OE1 (Subunit 0)	5.27
Phe1@N (Subunit 0) - Glu5@OE2 (Subunit -1)	7.06

PilA5

distance between Cα and N-terminus Cα (Angstrom):

amino acid

		E5	D26	D29	D37	D61	E68	D81	D93	C-terminus
	+2	>20	>20	>20	>20	>20	>20	>20	>20	>20
	+1	13.9	>20	>20	>20	>20	>20	>20	>20	>20
	0	6.6	>20	>20	>20	>20	>20	>20	>20	>20
	-1	11.6	>20	>20	>20	>20	>20	>20	>20	>20
	-2	>20	>20	>20	>20	>20	>20	>20	>20	>20
subunit	-3	>20	18.0	>20	>20	>20	>20	>20	>20	>20
	-4	>20	18.4	19.6	>20	>20	>20	>20	>20	>20
	-5	>20	>20	17.6	10.6	>20	13.0	16.6	>20	>20
	-6	>20	>20	>20	11.6	>20	16.8	>20	>20	>20
	-7	>20	>20	>20	>20	>20	>20	>20	>20	>20
	-8	>20	>20	>20	>20	>20	>20	>20	>20	>20
	-9	>20	>20	>20	>20	>20	>20	>20	>20	>20
	-10	>20	>20	>20	>20	>20	>20	>20	>20	>20

distance between specified atoms (Angstrom):

Phe1@N (Subunit 0) - Glu5@OE2 (Subunit 0)	4.62

Supplementary Table 3: Measured distances between the N-terminus and negatively charged side chains in PilA4 and PilA5

The distances between Cα of the N-terminal Phe and Cα (carboxyl-C for C-terminus) of negatively charged amino acids were measured for PilA4 (top) and PilA5 (bottom). For distances smaller than 10 Å (highlighted yellow in the table) the distance between the N-terminal nitrogen atom and the closest oxygen atom in the respective side chain were measured (shown underneath, atoms specified according to Chimera, lowest distance highlighted in green). For both filaments the closest negative charge to the N-terminus is Glu5 within the same subunit.

Peak	Pili OHy							
	Average GU	Composition	1	2	3	Average	STDev	CV
			% Area	% Area	% Area	% Area		
1	1.71	* W-H-PROC	7.69	1.80	2.93	4.14	3.13	75.5%
2	1.80	W-H-(Ac1)N-PROC	1.02	0.73	0.83	0.86	0.14	16.8%
3	1.84	(Ac1)W-H-N-PROC	0.65	0.47	0.53	0.55	0.09	16.5%
4	1.94	W-(Ac1)H-N-PROC	1.16	1.14	1.21	1.17	0.04	3.3%
5	2.05	W-H-(Ac1)N-(Ac1)N-PROC	0.64	-	0.37	-	0.19	-
6	2.31	?	0.54	0.51	0.62	0.56	0.06	10.3%
7	2.36	W-H-N-PROC	0.27	-	0.19	-	-	-
8	2.42	W-H-N-PROC	10.60	7.37	8.49	8.82	1.64	18.6%
9	2.48	W-H-N-(Ac1)N-PROC	2.45	3.39	3.08	2.98	0.48	16.1%
10	2.55	Ac1(X-H-N)-PROC	1.54	0.92	1.17	1.21	0.31	25.7%
11	2.57	(Ac1)W-H-N-N-PROC<br>Ac1(X-H-N)-PROC	1.89	2.03	2.02	1.98	0.08	4.0%
12	2.65	* X-H-PROC	8.24	3.44	4.32	5.33	2.55	47.8%
13	2.71	W-H-(Ac1)N-N-PROC	1.35	1.68	1.52	1.52	0.17	10.9%
14	2.78	Ac1(X-H-N)-PROC	0.79	0.58	0.62	0.67	0.11	16.5%
15	2.86	Y-H-N-PROC<br>(Ac1)X-H-(Ac1)N-N-PROC	0.44	-	0.17	-	-	-
16	2.90	?	0.48	-	0.26	-	-	-
17	2.94	W-H-(Ac1)N-PROC	0.24	-	0.15	-	-	-
18	3.05	W-H-N-PROC<br>X-H-N-(Ac1)N-PROC	2.48	3.34	3.02	2.95	0.43	14.7%
19	3.11	W-H-N-N-PROC	21.51	30.98	27.62	26.70	4.80	18.0%
20	3.17	X-H-N-PROC	12.11	8.57	9.69	10.12	1.81	17.8%
21	3.25	X-H-(Ac1)N-N-PROC	1.13	1.57	1.44	1.38	0.23	16.4%
22	3.32	?<br>W-H-(H)N-PROC	0.61	0.92	0.82	0.78	0.16	20.3%
23	3.44	X-H-(Ac1)N-N-PROC<br>W-H-(Ac1)N-PROC	1.08	1.54	1.40	1.34	0.23	17.4%
24	3.49	?	0.23	0.25	0.26	0.25	0.02	7.0%
25	3.67	W-H-N-N-PROC	0.56	0.82	0.75	0.71	0.13	18.9%
26	3.74	-	0.20	-	0.25	-	-	-
27	3.79	X-H-N-N-PROC	19.01	26.71	24.54	23.42	3.97	17.0%
28	3.84	X-H-N-N-PROC	0.51	0.48	0.57	0.52	0.05	8.8%
29	3.91	X-H-(H)N-PROC	0.30	0.32	0.38	0.33	0.04	12.9%
30	3.98	X-H-N-N-PROC	0.30	0.42	0.40	0.37	0.06	16.9%

Supplementary Table 4: Summary of average GU values, relative % areas and possible structures from O-glycans released from wild-type pili.

Peaks which produced CVs ≤ 20% are highlighted in green. Peaks which produced CVs ≥ 20% are highlighted in purple. OHy, O-mode hydrazinolysis; CV, coefficient of variation; GU, glucose unit; H, hexose; N, N-acetylhexosamine; W, pseudaminic acid derivative 5Am7Ac; X, unknown sugar 1 (346 Da); Y, unknown sugar 2 (330 Da); Ac = acetylation; question marks

label glycans with unassigned structure due to insufficient MS/MS data. Degradation products of the hydrazinolysis release (peeling products) are labelled with stars.

Source data are provided in a Source Data file.
