# Supplementary material for: Cryo-electron microscopy reveals two distinct type IV pili assembled by the same bacterium
Source: Nat Commun. 2020 May 6;11:2231. doi: 10.1038/s41467-020-15650-w (PMC7203116; doi:10.1038/s41467-020-15650-w)
Supplement: Supplementary file 3 — Reporting Summary [file 41467_2020_15650_MOESM3_ESM.pdf]

nature research

NCOMMS-19-30680-T
Corresponding author(s): Vicki Gold
Last updated by author(s): Mar 11, 2020

Reporting Summary

Nature Research wishes to improve the reproducibility of the work that we publish. This form provides structure for consistency and transparency in reporting. For further information on Nature Research policies, see Authors & Referees and the Editorial Policy Checklist .

Statistics

For all statistical analyses, confirm that the following items are present in the figure legend, table legend, main text, or Methods section.

n/a	Confirmed	
☐	☒	The exact sample size (*n*) for each experimental group/condition, given as a discrete number and unit of measurement
☐	☒	A statement on whether measurements were taken from distinct samples or whether the same sample was measured repeatedly
☐	☒	The statistical test(s) used AND whether they are one- or two-sided<br>*Only common tests should be described solely by name; describe more complex techniques in the Methods section.*
☒	☐	A description of all covariates tested
☒	☐	A description of any assumptions or corrections, such as tests of normality and adjustment for multiple comparisons
☒	☐	A full description of the statistical parameters including central tendency (e.g. means) or other basic estimates (e.g. regression coefficient) AND variation (e.g. standard deviation) or associated estimates of uncertainty (e.g. confidence intervals)
☒	☐	For null hypothesis testing, the test statistic (e.g. *F*, *t*, *r*) with confidence intervals, effect sizes, degrees of freedom and *P* value noted<br>*Give P values as exact values whenever suitable.*
☒	☐	For Bayesian analysis, information on the choice of priors and Markov chain Monte Carlo settings
☒	☐	For hierarchical and complex designs, identification of the appropriate level for tests and full reporting of outcomes
☒	☐	Estimates of effect sizes (e.g. Cohen's *d*, Pearson's *r*), indicating how they were calculated

Our web collection on statistics for biologists contains articles on many of the points above.

Software and code

Policy information about availability of computer code

Data collection	Digital Micrograph version 3.32.2403.0<br>EPU version 1.10<br>Xcalibur version 2.1<br>Chromeleon version 7.2.2
Data analysis	IMOD version 4.10.11<br>UNBLUR version 1.0.2<br>CTFFIND version 4.1.5<br>RELION version 3.0.8<br>EMAN2 version 2.12<br>SPRING version 0.84<br>Chimera version 1.11<br>ChimeraX version 0.6<br>ISOLDE version 1.0b1<br>Coot version 0.9-pre<br>Refmac5 version 5.8.0158<br>Fiji: ImageJ version 1.51h<br>Kappa version 1.5.6<br>MaxQuant version 1.5.3.8<br>Perseus version 1.5.2.6<br>Proteome Discoverer 1.4

For manuscripts utilizing custom algorithms or software that are central to the research but not yet described in published literature, software must be made available to editors/reviewers. We strongly encourage code deposition in a community repository (e.g. GitHub). See the Nature Research guidelines for submitting code & software for further information.

Data

Policy information about availability of data

All manuscripts must include a data availability statement. This statement should provide the following information, where applicable:

- Accession codes, unique identifiers, or web links for publicly available datasets
- A list of figures that have associated raw data
- A description of any restrictions on data availability

EM maps have been deposited in the Electron Microscopy Data Bank (EMDB, https://www.ebi.ac.uk/pdbe/emdb/) with the accession codes EMD-10647 (wide pilus, PilA4) and EMD-10648 (narrow pilus, PilA5). Models have been deposited in the Protein Data Bank (PDB, https://www.rcsb.org/) with accession codes 6XXD (PilA4) and 6XXE (PilA5). The MS proteomics data have been deposited to the ProteomeXchange Consortium via the PRIDE partner repository (https://www.ebi.ac.uk/pride/) with the dataset identifier PXD017353. The source data underlying Figure 5c-e, Figure 6, Supplementary Figure 2, Supplementary Figure 3b,c, Supplementary Figure 4a-c, Supplementary Figure 5e, Supplementary Figure 6e, f and Supplementary Table 4 are provided in a Source Data file. Uncropped versions of gels and blots (Figure 5c, d, Supplementary Figure 2b, Supplementary Figure 4a-c) and twitching images (Figure 6a) are also shown in Supplementary Figure 10.

Field-specific reporting

Please select the one below that is the best fit for your research. If you are not sure, read the appropriate sections before making your selection.

☒ Life sciences ☐ Behavioural & social sciences ☐ Ecological, evolutionary & environmental sciences

For a reference copy of the document with all sections, see nature.com/documents/nr-reporting-summary-flat.pdf

Life sciences study design

All studies must disclose on these points even when the disclosure is negative.

Sample size	No specific statistical methods were used to determine sample size as it was not generally applicable to our study. For all quantitative analysis, gels and blots, all available data points were used and confirmed initial findings. For electron microscopy data, sample size was determined by the availability of areas to image on grids.
Data exclusions	No data have been excluded
Replication	For quantitative experiments (Figure 6b and Supplementary Figure 2a), n = 4. Gels and blots (Figure 5c, d, Supplementary Figure 2b, Supplementary Figure 4a-c) were all n = 3 (one gel was used for subsequent MS in Supplementary Figure 2b) and in all cases similar findings were reported. Twitching experiments (Figure 6a), n = 3. Electron microscopy data (Supplementary Figure 3) are representative from >100 images and the findings are described by quantitative data (Supplementary Figure 3b, c), n = 3. Electron microscopy data (Supplementary Figure 5) are representative from >1000 images and the findings are described by quantitative data (Supplementary Figure 5e), n = 3. In Figure 5e, n= 3 for UHPLC and subsequently one of the samples (Pili wt) was used for MS. For tomography data of cells (Figure 1), n = 35 tomograms; 20 T4P complexes were assigned to wide or narrow pilus groups, representative images are shown. For tomography data of pili (Supplementary Figure 1), n = 9 tomograms; >50 pili were assigned to wide or narrow pilus groups, representative images are shown. Data shown in Figure 2, 3, 4, 5a, b, Supplementary Figure 6 and Supplementary Figure 9 are based on two independently determined cryoEM maps from a single data set of 3,138 images.
Randomization	Cells for Supplementary Figure 3b,c were selected at random based on low magnification overview micrographs. All pili were counted. Micrographs for Supplementary Fig. 5e and tomograms for Fig. 1 and S1 were selected at random.
Blinding	Blinding was not relevant since no data was excluded and possible biased evaluation steps were calculated using established software.

Reporting for specific materials, systems and methods

We require information from authors about some types of materials, experimental systems and methods used in many studies. Here, indicate whether each material, system or method listed is relevant to your study. If you are not sure if a list item applies to your research, read the appropriate section before selecting a response.

Materials & experimental systems

n/a	Involved in the study	
☐	☒	Antibodies
☒	☐	Eukaryotic cell lines
☒	☐	Palaeontology
☒	☐	Animals and other organisms
☒	☐	Human research participants
☒	☐	Clinical data

Methods

n/a	Involved in the study	
☒	☐	ChIP-seq
☒	☐	Flow cytometry
☒	☐	MRI-based neuroimaging

Antibodies

Antibodies used	polyclonal PilQ antibodies
Validation	PilQ antibodies are described in Rumszauer et al. (2006) doi:10.1111/j.1742-4658.2006.05335.x
